# High-Intensity Focused-Ultrasound Focal Therapy Versus Laparoscopic Radical Prostatectomy: A Comparison of Oncological and Functional Outcomes in Low- and Intermediate-Risk Prostate Cancer Patients

**DOI:** 10.3390/jpm12020251

**Published:** 2022-02-09

**Authors:** Łukasz Nyk, Wojciech Michalak, Stanisław Szempliński, Rafał Woźniak, Bartłomiej Zagożdżon, Wojciech Krajewski, Piotr Kryst, Hubert Kamecki, Sławomir Poletajew

**Affiliations:** 1 Second Department of Urology, Centre of Postgraduate Medical Education, 01-809 Warsaw, Poland; lukasz.nyk@cmkp.edu.pl (Ł.N.); wojciech.michalak@ecz-otwock.pl (W.M.); stanislaw.szemplinski@cmkp.edu.pl (S.S.); bartlomiej.zagozdzon@ecz-otwock.pl (B.Z.); piotr.kryst@bielanski.med.pl (P.K.); slawomir.poletajew@cmkp.edu.pl (S.P.); 2 Chair of Statistics and Econometrics, Faculty of Economic Sciences, University of Warsaw, 00-241 Warsaw, Poland; rwozniak@wne.uw.edu.pl; 3 Department of Minimally Invasive and Robotic Urology, University Center of Excellence in Urology, Wrocław Medical University, 50-556 Wrocław, Poland; wojciech.krajewski@umw.edu.pl

**Keywords:** prostate cancer, focal therapy, high-intensity focused-ultrasound, urinary incontinence, erectile dysfunction

## Abstract

To compare oncological and functional outcomes of high-intensity focused-ultrasound (HIFU) focal therapy (FT) versus laparoscopic radical prostatectomy (LRP) in patients treated for low- or intermediate-risk prostate cancer (PCa), we retrospectively analyzed data of consecutive patients comprising 30 men, who underwent HIFU-FT, and 96 men who underwent LRP, in an academic center. Oncological outcomes were assessed based on the follow-up prostate-specific antigen values. We used the International Index of Erectile Function short form score to assess erectile function (EF). Urinary continence status was defined based on the number of pads used per day. Median follow-up was 12.5 and 19.1 months in the LRP and HIFU-FT groups, respectively. The effects were computed after propensity score matching and expressed as average treatment effect (ATE). Compared to LRP, HIFU-FT was associated with increased risk of treatment failure (ATE 0.103–0.164, depending on definition, *p* < 0.01) and lower risk of urinary incontinence (ATE −0.808 at 12 months, *p* < 0.01). Risk of erectile dysfunction was higher in the LRP group (ATE 5.092, *p* < 0.01). Our results demonstrate that HIFU-FT may be a reasonable treatment option in selected PCa patients, willing to preserve their EF and urinary continence yet accepting a higher risk of treatment failure.

## 1. Introduction

Prostate cancer (PCa) is one of the most prevalent malignancies in the world. As reported by the World Health Organization (WHO), over 1.4 million new cases were diagnosed worldwide in 2020, which translate to 36 new diagnoses per 100,000 men [1]. Surprisingly, the mortality rates of PCa seem less appalling and are over three times lower than in lung cancer.

PCa is a very heterogeneous disease and its clinical course may vary. Patients with aggressive, poorly differentiated cancers may experience rapid metastatic spread and death despite early implementation of systemic treatment. Others, diagnosed with more indolent forms may never progress, remaining stable in observation without undergoing any treatment at all. Therefore, according to the European Association of Urology (EAU) Guidelines, every patient should receive treatment adapted to their stage and grade [2]. Active surveillance is the strategy of choice in low-risk patients, whereas those with organ-confined disease but at higher risk of progression should undergo radical treatment.

However, some men diagnosed with low- to intermediate-risk PCa are either reluctant to undergo surveillance or unwilling to accept the potential side effects and complications of radical treatment. Focal therapy (FT) was developed as a novel modality aimed at answering their needs, potentially allowing the patient to undergo active treatment while decreasing their risk of functional outcomes being compromised. Unfortunately, the use of FT remains controversial, as no reliable data from randomized controlled trials assessing its oncological efficacy are available. Thus, the purpose of our study was to compare the oncological and functional results of FT to the outcomes of patients treated with laparoscopic radical prostatectomy (LRP).

## 2. Materials and Methods

We retrospectively analyzed the data of consecutive patients who underwent high-intensity focused-ultrasound (HIFU) FT or LRP in our institution between 2016 and 2019. The inclusion criteria were as follows: (i) low or intermediate risk as per EAU risk group definition (T1c–T2b, grade group 1–3, prostate-specific antigen (PSA) ≤ 20 ng/mL), (ii) not having undergone prior treatment for PCa, (iii) Charlson Comorbidity Index ≤ 4 at surgery (not including the points for prostate solid tumor), (iv) Eastern Cooperative Oncology Group (ECOG) score at surgery equal to 0.

### 2.1. Treatment Methods

All the low-risk PCa patients who received active treatment had refused to undergo active surveillance. All the PCa patients eligible for active treatment had been offered radiotherapy before proceeding with LRP or HIFU-FT.

All patients who underwent HIFU-FT had been initially diagnosed with multiparametric magnetic resonance imaging (mpMRI) and prostate biopsy. Potential treatment modalities had been discussed with each patient, and FT had been offered only to those highly motivated to actively treat their disease but unwilling to undergo any form of radical treatment. If the calculated probability of lymph node (LN) invasion, as assessed with the Briganti nomogram [3], had exceeded 5%, the patient would be informed about the risk and would undergo HIFU-FT only if refusing to undergo LRP with extended LN dissection (eLND).

Each focal procedure was performed with a Sonablate 500 3G device (manufacturer: Sonablate Corp., Charlotte, NC, USA). Under general anesthesia, the patient was placed in the lithotomy position and a Foley catheter was inserted into the bladder. The HIFU probe was then installed into the rectum, in the vicinity of the prostate. Next, the treatment was meticulously planned and the procedure was launched. Depending on the extent of the cancer focus, either a focal ablation of the index lesion together with a 5 mm safety margin or hemiablation of the prostate was performed.

In the control group, LRP was performed with either extra- or transperitoneal approach, depending on the probability of lymph node invasion, as assessed with the Briganti nomogram [3]. If the calculated risk had exceeded 5%, the patient underwent transperitoneal surgery with extended lymphadenectomy. Uni- or bilateral neurovascular bundle preservation was performed whenever possible, depending on tumor location and characteristics. All LRPs were performed with the intention of bladder neck preservation and single-knot method with 4–0 running barbed suture used for urethrovesical anastomosis.

### 2.2. Oncological Outcomes

After the treatment, all patients were followed up with clinical visits and PSA testing every 3 months during the first postoperative year and every 6 months thereafter.

In the HIFU-FT group, optional use of follow-up mpMRI was at the attending urologist discretion. A prostate biopsy was performed in case of a rise in PSA or signs of treatment failure on mpMRI.

Three independent definitions of treatment failure in the HIFU-FT group were used in this study. The first definition was adopted from whole-gland HIFU treatment: the Stuttgart criteria [4] define biochemical recurrence (BCR) as a PSA increase of >1.2 ng/mL above the nadir. According to the second definition, failure was defined as either BCR or a positive prostate biopsy during follow-up. The third definition perceived failure as a positive biopsy during follow-up, regardless of the indications that triggered the biopsy; for the purpose of this study, a biopsy was considered positive only in cases when cancer tissue was present in the cores taken from the treated zone.

Treatment failure in the LRP group was defined as either a postoperative or a follow-up PSA level being above 0.2 ng/mL.

### 2.3. Functional Outcomes

All patients answered the International Index of Erectile Function short form (IIEF-5) questionnaire at baseline and at 12 months after surgery. The difference in the change in the IIEF-5 scores were used to compare the two groups.

Urinary continence status was defined based on the number of pads used per day at 3 and 12 months. Full continence status was defined as use of 0 pads per day. The difference in the number of pads used and the difference in the full continence rate were used to compare the two groups.

### 2.4. Statistical Analysis

To reduce a possible bias caused by the imbalance in patient characteristics, we performed propensity-score matching (PSM) prior to comparing outcomes. The PSM was performed in a 1:1 ratio, using the following variables: age, baseline PSA level, prostate volume, grade group at preoperative biopsy, baseline IIEF-5 score, preoperative clinical stage at digital rectal examination (DRE, cT), and preoperative clinical stage at mpMRI (iT).

To express the results, we used average treatment effect (ATE), which translates to the expected effect of treatment for a randomly drawn individual. Alongside this, we calculated average treatment effect on treated (ATET), which describes the expected effect of treatment for the treated individuals.

Kaplan–Meier curves presenting differences in failure-free survival were drawn for all three definitions of FT failure.

The results were considered statistically significant if *p* value < 0.05.

## 3. Results

Thirty patients who underwent HIFU-FT and 96 who underwent LRP met the inclusion criteria. Patient characteristics are presented in Table 1. Median follow-up was 12.5 months in the LRP patients and 19.1 months in the FT group.

### 3.1. Oncological Outcomes

#### 3.1.1. High-Intensity Focused Ultrasound-Focal Therapy (HIFU-FT) Treatment Failure Defined as a Prostate-Specific Antigen (PSA) Rise >1.2 ng/mL above the Nadir Only

Using this first definition, failure occurred in 7 (23.33%) patients treated with FT, compared to 0 (0%) undergoing LRP. We found that men treated with FT had a 10.3% higher probability of failure than those in the control group (ATE = 0.103, *p* < 0.01). Our analysis also revealed that if they had undergone LRP instead, this risk would have been reduced by 23.3% (ATET = 0.233, *p* < 0.01).

#### 3.1.2. HIFU-FT Treatment Failure Defined as Either PSA Rise > 1.2 ng/mL or a Positive Biopsy during Follow-Up

According to the second definition, failure occurred in 10 (33.3%) patients treated with FT, compared to 0 (0%) undergoing LRP. In this case, men treated with FT had a 16.4% higher probability of failure than those in the control group (ATE = 0.164, *p* < 0.01). If they had have received LRP instead, this risk would have been reduced by 33.3% (ATET = 0.333, *p* < 0.01).

#### 3.1.3. HIFU-FT Treatment Failure Defined as a Positive Biopsy Only

Under the third definition, failure occurred in 8 (27.59%) patients treated with FT, compared to 0 (0%) undergoing LRP. Men treated with FT had an 11.3% higher probability of failure than those in the control group (ATE = 0.113, *p* < 0.01). If they had been treated with LRP instead, their risk would have been reduced by 27.6% (ATET = 0.276, *p* < 0.01).

**Figure 1:**
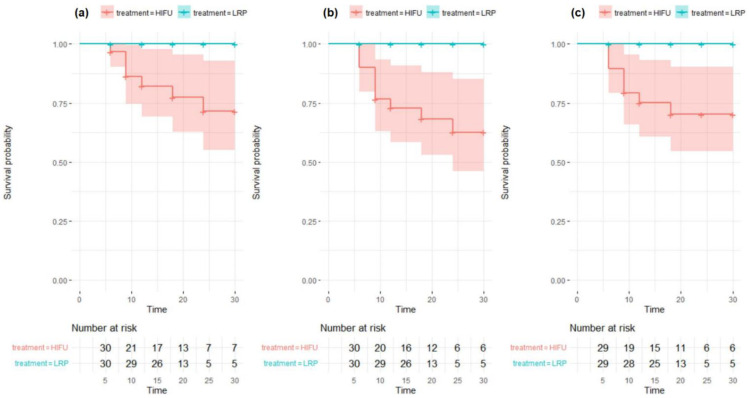
Kaplan–Meier curves presenting differences in failure-free survival between the highly-intensive focused-ultrasound (HIFU) and laparoscopic radical prostatectomy (LRP) groups: (**a**) treatment failure (TF) defined as a prostate-specific antigen (PSA) rise >1.2 ng/mL above the nadir, (**b**) TF defined as either PSA rise >1.2 ng/mL above the nadir or positive biopsy on follow-up, (**c**) TF defined as positive biopsy on follow-up.

The Kaplan–Meier curves demonstrating the differences in failure-free survival between HIFU-FT and LRP groups are presented in Figure 1.

### 3.2. Urinary Continence

When determining urinary continence status, we compared the absolute numbers of pads used by patients at 3 and 12 months after treatment. At both time points the difference was significant and favored focal therapy (1.80 and 0.43 pads at 3 and 12 months, respectively, which translates to ATE −2.093 and −0.808, respectively, *p* < 0.01). We also discovered that HIFU-FT patients had a 90% lower probability of not being fully continent (defined as using any pads) during follow-up (ATE −0.900, *p* < 0.01).

### 3.3. Erectile Function

The absolute difference between IIEF-5 score at baseline and at 12 months post-treatment was used as the determinant of the impact of a treatment modality on erectile function. We estimated that the change in IIEF-5 was higher in the LRP group (7.40 versus 0.03 points in the HIFU-FT group). The ATE amounted to 5.092, which means that performing focal therapy altered IIEF-5 by 5.092 points less than LRP did (*p* < 0.01).

## 4. Discussion

Focal therapy is a PCa treatment strategy aimed at direct ablation of predefined cancer lesions in the prostate while leaving the rest of the gland untreated. The wide adoption of mpMRI in treatment-naive patients in recent years has led to more accurate detection of cancer foci and more careful patient selection [5]. According to the current literature, FT provides good cancer control [6,7,8] and is associated with excellent functional outcomes [7,8,9]. The rates of severe complications also seem to be lower than those of radical treatment modalities [10]. Several energy sources have been used to date (high-intensity focused ultrasound, cryotherapy, irreversible electroporation etc.) [11,12,13,14,15,16], but no reliable data favoring one source over another exist [17].

Although FT is promising, several controversies have arisen. First, no data from randomized controlled trials comparing its oncological efficacy with radical treatment are available. Second, no agreement exists on what really determines the focality of the therapy [9,16]. Historically, the name FT was used for the treatment of both uni- and bilateral disease. In unilateral and unifocal cases, some clinicians tend to perform hemiablation of the prostate, whereas others restrict ablation to predefined cancer lesions. In unilateral and multifocal disease, hemiablation is also widely used, yet focal destruction of all known cancer foci or ablating only the so-called index lesion has gained popularity. The same discrepancies apply to bilateral cancer cases. No data favoring one strategy over another exist. When deciding to target the lesion, the clinician needs to define the safety margin surrounding the cancer tissue, but no reliable recommendations are available. This uncertainty is disturbing, as it seems likely that the range of ablation may alter both the efficacy and toxicity of the method. At our facility, we perform either focal ablation of the index lesion together with a 5 mm safety margin or hemiablation in the case of bigger cancer foci; however, no particular cut-off point is used, and the decision depends solely on surgeons’ judgment.

Furthermore, treating only a part of the gland makes the follow-up more challenging. In contrast to radical prostatectomy, the levels of post-treatment PSA after FT are very hard to predict, and a rise does not necessarily imply failure. Many different definitions of BCR after FT have been proposed, however none of them was validated or deemed appropriate [9]. Although ideal biochemical criteria are yet to be determined, in everyday practice PSA testing remains an important issue. Two cut-off points are widely used for focal therapy: the Phoenix criterion (rise > 2 ng/mL over nadir) and the Stuttgart criterion (rise > 1.2 ng/mL over nadir). As shown by Kongnyuy et al., both may be used to determine BCR in PCa patients who underwent focal therapy [18].

Since treatment failure cannot be diagnosed solely with PSA testing, many experts define it as a positive prostate biopsy during follow up. It is generally agreed that only the presence of cancer tissue in cores from the treated zone implies true ineffectiveness of the procedure. However, whether the biopsy should be performed on a regular basis at a particular time point or only be triggered by a suspicious PSA level or mpMRI findings remains a matter of dispute. According to an international expert consensus meeting, persistence of grade group 1 cancer in the treated zone with a core length ≤3 mm is clinically acceptable and not considered treatment failure if the primary lesion was of a higher grade or a higher volume prior to the intervention [19]. Not all clinicians performing FT agree with this statement.

Given the lack of clear definition of treatment failure, we believe that the follow-up of patients after FT should not be based on a single variable and should preferably combine PSA kinetics monitoring with mpMRI and biopsy [20]; however, it is evident that establishing an optimal protocol will require further studies.

In our study, we found that HIFU-FT was inferior to LRP in terms of cancer control. However, it was associated with much better preservation of urinary continence, as well as erectile function, even despite nerve-sparing was performed in 90% of LRP patients (after PMS). The outcomes were consistent across all tested failure definitions and corresponded with those recently presented by Garcia-Barreras [21]. However, it is worth mentioning that while all of our patients underwent pure LRP, performing robot-assisted LRP instead could have led to better functional results, as suggested by the literature [22,23,24], which would have diminished the observed superiority of HIFU-FT.

Interestingly, although four patients experienced biochemical failure after radical prostatectomy, no case of failure was found in the LRP group after PSM. According to contemporary guidelines, patients with organ-confined disease and long life-expectancy are offered either active surveillance (AS) or radical treatment, depending on their disease stage and grade. Unfortunately, some men are either not eligible for or reluctant to undergo AS but at the same time they do not accept the potential side effects and complications of radical treatment. Focal therapy may be an option for them. Our data suggest that FT may be considered inferior to radical prostatectomy as far as treatment failure rates are concerned; however, observations based on bigger patient cohorts, published by Shah and Albissini, show that the oncological outcomes may actually be similar to radical treatment [6,8]. Furthermore, the occurrence of failure after FT does not preclude successful treatment. Second-line FT is considered feasible and acceptable if the retreatment rates do not exceed 20%. The oncologic outcomes of salvage radical treatment after FT seem comparable with radical treatment performed in the primary setting [25]. Iatrogenic periprostatic fibrosis may be considered a potential obstacle when considering salvage surgical management in patients who had previously underwent HIFU-FT. However, a study by Marconi et al. revealed, that salvage robot-assisted laparoscopic prostatectomy (RALP) after focal therapy results in no increase in toxicity when compared to primary RALP [26]. On the other hand, Spitznagel et al. reported higher rates of Clavien-Dindo III complications in patients undergoing salvage RALP after HIFU-RT in comparison to primary RALP patients [27]. Nevertheless, in our opinion, focal therapy remains an interesting treatment option for selected patients with organ-confined prostate cancer. Thorough staging, wise case selection, and the provision of detailed information to the patients seem crucial to success.

Important advantages of our study are the use of ATE and ATET to illustrate treatment outcomes and the presentation of the oncological results of focal therapy under three independent definitions of failure. The major limitation is the retrospective design of the study. Several baseline patient characteristics could have influenced both the treatment selection, as well as observed outcomes, and while this could be avoided in a prospective trial, the lack of designed patient selection and randomization poses a non-negligible risk of bias to our results. Propensity score matching was employed to partially overcome this limitation. Other important limitations include small sample size, relatively short follow-up, and non-adjusting for surgeon experience when analyzing the functional outcomes.

## 5. Conclusions

In our study, HIFU-FT was associated with a higher rate of treatment failure than laparoscopic radical prostatectomy. However, it provided significantly better functional outcomes. Based on the presented results, we believe that HIFU-FT remains a viable treatment option in selected patients motivated to preserve their erectile function and urinary continence yet accepting a slightly increased risk of compromised oncologic results.

## Figures and Tables

**Table 1:** Patient characteristics.

Parameter	HIFU-FT (*n* = 30)	LRP, Prior to PSM (*n* = 96)	LRP, After PSM (*n* = 30)
Median age (years)	64.5	65.7	64.0
Mean PSA (ng/mL)	6.6	6.6	6.5
Mean PV (mL)	38.0	42.6	39.4
Mean baseline IIEF-5 score	19.2	14.7	19.9
Median follow-up (months)	19.1	10.4	12.5
cT1c ^a^	17 (57%)	63 (66%)	16 (53%)
cT2a ^a^	12 (40%)	14 (15%)	6 (20%)
cT2b ^a^	1 (3%)	19 (20%)	8 (27%)
Grade group 1	20 (67%)	63 (66%)	18 (60%)
Grade group 2	9 (30%)	33 (34%)	12 (40%)
Grade group 3	1 (3%)	0 (0%)	0 (0%)
Nerve-sparing			
any	N/A	79 (82%)	27 (90%)
bilateral	N/A	59 (61%)	25 (83%)
Intrafascial ^b^	N/A	47 (49%)	20 (67%)
Median hospital stay (days)	2.0	3.6	3.7

HIFU-FT—high-intensity focused ultrasound-focal therapy; LRP—laparoscopic radical prostatectomy; PSM—propensity score matching; PSA—prostate-specific antigen; PV—prostate volume; IIEF-5—international index of erectile function-5; N/A—non-applicable; ^a^ the cT1c–2b stages correspond to the clinical assessment at DRE. ^b^ intrafascial dissection performed at least unilaterally.

## Data Availability

The data analyzed in this study are available upon request from the corresponding author.
